# Axial and Radial Spatial Patterns of Non-Structural Carbohydrates in *Cycas micronesica* Stems

**DOI:** 10.3390/plants7030049

**Published:** 2018-06-22

**Authors:** Thomas E. Marler

**Affiliations:** College of Natural and Applied Sciences, University of Guam, UOG Station, Mangilao, GU 96923, USA; thomas.marler@gmail.com; Tel.: +1-671-735-2100

**Keywords:** cycad, fructose, glucose, maltose, sucrose, starch

## Abstract

The pachycaulous stem of arborescent cycad species exhibits unique traits and has received limited research. To date, nothing is known about the axial and radial spatial patterns of non-structural resources within cycad stems. *Cycas micronesica* K.D. Hill stem tissue was collected from apical and basal axial positions of ca. 100-cm tall plants to serve as two axial regions; and from pith, vascular, and cortex tissues to serve as three radial regions. Starch and four free sugars were quantified. These stems contained more starch than any of the individual sugars, and sucrose concentration exceeded that of fructose and glucose, which exceeded that of maltose. Total non-structural carbohydrate was least in basal vascular tissue (225 mg·g^−1^) and greatest in apical pith tissue (379 mg·g^−1^). Axial differences in NSC concentrations were negligible but radial differences were substantial. These results combine with past research to validate the non-woody cycad stem contains copious nonstructural resources available for deployment to ephemeral sinks during critical times of need.

## 1. Introduction

*Cycas micronesica* is an arborescent cycad species from several island groups in Micronesia [1]. The tree is one of 350 cycad species [2] that comprise the most threatened group of plants worldwide [3,4]. The cycad pest *Aulacaspis yasumatsui* Takagi (Cycad Aulacaspis Scale (CAS)) invaded Guam in 2003 [5,6], and this invasion was the start of several biological threats that have caused widespread mortality of the tree species [7]. The extent of mortality generated a 2006 Endangered listing under the International Union for Conservation of Nature and Natural Resources (IUCN) [8] and a 2015 Threatened listing under the United States Endangered Species Act [9].

The use of large *C. micronesica* stem cuttings as propagules was evaluated to determine if the approach could be employed to rescue trees from construction sites on Guam [10]. Low to moderate success rates were realized due to the compromised health status of the CAS-infested trees. The findings indicated that more research to determine the potential causes of limited propagation success was required before implementing the procedure on a large scale [11]. Toward that end, *Cycas revoluta* Thunb. were experimentally infested with CAS in controlled conditions, and the herbivore caused lethal depletions of non-structural carbohydrates (NSC) [12]. Moreover, in situ *C. micronesica* trees were experimentally protected from ubiquitous CAS, and the increases in NSCs following protection from CAS herbivory were correlated with increased asexual propagation success of stem cuttings [13].

**Figure 1:**
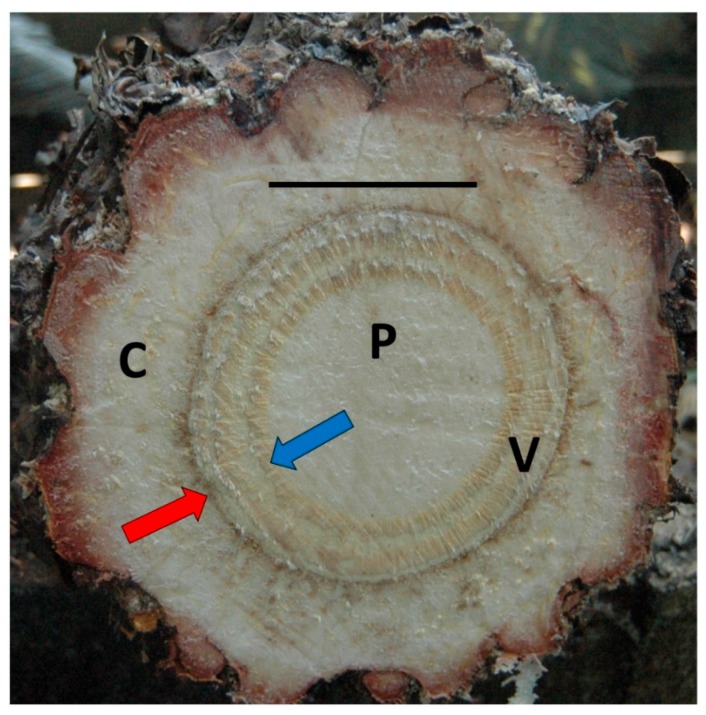
Internal structure of an arborescent *Cycas* stem shows characteristic live tissues among pith (P), vascular cylinders (V), and persistent cortex (C) radial regions. Blue arrow is oldest vascular cylinder, red arrow is youngest vascular cylinder. Horizontal bar is 10 cm.

The current CAS outbreak within the native range of *C. micronesica* motivated my study of terrestrial plant conservation issues to identify critical areas of limited knowledge. Considering the importance of NSCs to host plant survival following CAS infestation, more research is clearly needed to fully understand NSC relations of damaged trees and how these resources relate to tree mortality. One facet of NSC relations of cycads that has not been determined is the spatial dynamics of stored NSC within the stem. Cycad stems contain persistent cortex that is peripheral to concentric vascular cylinders, and these cylinders conceal a large living pith stem center (Figure 1) [14]. These traits illuminate how cycad stems are unique in design and are comprised almost exclusively of living tissues. Therefore, the axial and radial stratification of NSCs within woody tree stems may have minimal relevance for predicting spatial dynamics of cycad stem resources. My objective was to determine the distribution of NSCs within *C. micronesica* stems among three radial zones and two axial zones.

## 2. Materials and Methods

Ten healthy container-grown *Cycas micronesica* plants were used in May 2016 to obtain tissue samples from six stem locations. The plants were grown from seeds derived from cultivated material that was originally from southern Guam, and the six-year-old plants had been grown in container medium that was roughly equal parts of peat:perlite:sand:field soil since germination. Container size was 2.6 L initially, and the plants were transferred to larger containers as needed such that the final container size was 15.5 L. The plants were grown underneath 50% shade screen for the first 3 years, and under full sun conditions thereafter. Rainfall was augmented with hand-watering as needed. A granular fertilizer (12N–2.6P–6.6K plus micronutrients) was provided to each container approximately every 8 weeks at the rate of 5 g initially and 30 g for the final container size. In addition, a 125-mL drench of 2.5 mM Fe (as Fe-EDDHA) solution was applied to each container every 8 weeks. Protection from CAS was provided by root application of imidacloprid every 2–3 months. Total plant height of the monopodial plants ranged from 150 to 175 cm, and stem height ranged from 105 to 110 cm.

A 2-cm disc was cut from the apical region of the stems directly below the leaves, where stem diameter ranged from 12 to 16 cm. Similarly, a 2-cm disc was cut from the base of the stems just above the root collar, where stem diameter ranged from 12 to 15 cm. The axial position of this basal disc was standardized to 100 cm below the oldest leaf position. These two axial positions will be called apical and basal hereafter. Each disc was separated into three sections. The peripheral section was the cortex, and no attempt was made to remove the dermal tissue from the living cortex tissues. The intermediate section was comprised of the concentric cylinders of vascular tissue. All vascular tissues were combined into one composite sample. The innermost section was the pith. These three radial positions will be called cortex, vascular, and pith sections hereafter.

The tissue was immediately frozen at −20 °C, lyophilized, and milled to pass through a 20-mesh screen. Soluble sugar extraction was conducted using hot-water extraction with acetonitrile (80 °C) [15]. The concentrations of four free sugars (the hexoses fructose and glucose, and the disaccharides sucrose and maltose) were determined by HPLC-RI (Thermo Scientific RI-150, AS3000 autosampler, P2000 pump). Starch was quantified following hydrolyzation by amyloglucosidase to glucose [16].

The statistical analysis was conducted using SAS 9.3 (SAS Institute, Cary, Indiana) using a two-way ANOVA with two axial positions (apical and basal) and three radial positions (cortex, vascular, pith). Means separation was conducted by least significant difference for response variables that exhibited significant differences.

## 3. Results

### 3.1. Radial Differences in Resource Allocation

Four of the five NSCs did not exhibit an interaction between radial and axial *C. micronesica* stem sections but differed significantly among the radial tissue sections (Table 1). Fructose, glucose, maltose, and starch exhibited the lowermost concentrations in vascular tissues, with pith concentrations generally exceeding those of cortex. Total free sugar concentration followed the same pattern. The stoichiometric relationship between free sugars and starch was calculated as the quotient sugars/starch. This quotient ranged from 0.55 in vascular tissues to 0.88 in pith tissues. Therefore, starch was in greater concentration than sugars in all stem tissues, with pith tissues containing the most sugars relative to starch.

### 3.2. Interactions Among Axial and Radial Stem Sections

Sucrose and total NSC concentrations exhibited interactions between the axial and radial sources of variation of these *C. micronesica* stem sections (Table 2). Sucrose concentration was least in vascular and most in pith tissues in both axial locations. However, the behavior of sucrose in the cortex was influenced by the axial location such that concentration did not differ from that of vascular tissue in the stem apex and did not differ from that of pith tissue in the stem base. The pattern of total NSC followed that of sucrose for the stem base, but total NSC within the cortex was intermediate between pith and vascular tissues for stem apex.

## 4. Discussion

Structure and function of the pachycaulous cycad stem has been the subject of limited research interest [14,17,18,19,20,21]. Only few reports have detailed various components of the cycad stem NSC pool. The total NSC of 382 mg·g^−1^ reported for *C. revoluta* stems [12] and of 369 mg·g^−1^ reported for in situ *C. micronesica* stems [13] were remarkably similar to findings herein. Moreover, the total free sugar of 173 mg·g^−1^ for *C. revoluta* stems [12] and of 170 mg·g^−1^ reported for in situ *C. micronesica* stems [13] were also similar to the results herein. In contrast, the total free sugar of ca. 40 mg·g^−1^ reported for *Zamia muricata* Willd. stems [22] was much less than that of *C. micronesica* stems. Although the same methods were used among these studies, the limited number of species do not enable an interpretation of these contrasting results for the two genera. The differences, however, do illuminate the need for a robust comparison of NSC concentration and three-dimensional location among representatives of the described cycad taxa. Because methodological differences among studies can lead to contrasting results [23], pursuit of these answers would best be conducted with a single dedicated study rather than a series of ad hoc studies.

The general patterns of the NSCs in these *C. micronesica* stems indicated that radial variation was considerable and pith tissues at the center of the stem contained the greatest amounts of every NSC. For example, total free sugar concentration of pith tissues was almost double that of vascular tissues (Table 1). In contrast, 1 m of axial separation in stem position did not influence NSC concentration appreciably. The axial distribution of NSCs within stems of stressed trees may be controlled by distance from carbon source [24,25]. The upper stem location was as close to the leaves as possible, and the basal stem location was as far from the leaves as possible. Moreover, the basal sampling site was as close to the root sink activity as possible. Despite these spatial dynamics and proximity to separate source (apical) or sink (basal) organs, the differences in NSCs within these *C. micronesica* stems were minimal.

Radial and axial stem positions exhibited the general order of starch > sucrose > glucose > fructose > maltose. This NSC ranking is in agreement with that of healthy *Cycas revoluta* stem tissue [12] and in situ *C. micronesica* stem tissue [13].

These results validate that the cycad stem functions as a credible resource storage organ. The mean NSC concentrations reported here exceeded 300 mg·g^−1^, which were much greater than the 10–170 reported for stems of woody tree species [26,27,28,29,30,31,32]. Within a tropical forest, the 85–100 mg·g^−1^ range for stem NSC was also minimal compared to that reported herein [33]. Cycad stems are mostly living tissue, so these stem resources are available for mobilization and deployment to ephemeral sinks during plant development. As sessile, autotrophic, long-lived organisms, trees are required to persist through limitations of environmental resources, overt abiotic stresses, competition with neighboring plants, and herbivore or pathogen damage. In this light, the size of a tree’s NSC pool is a measure of its carbon fueling status [34], with stored NSC serving as reserves that the tree can mobilize in times of stress [35,36,37,38,39]. The vast storage of NSCs within the cycad stem is likely one of the reasons that cycads are known for their resilience following defoliation events such as tropical cyclones [40] and fires [41]. This readily available NSC pool within stems may also account for the highly successful use of small sub-apical cycad stem cuttings for asexual propagation of cycads [42].

The radial distribution of NSCs in the trunks of numerous woody tree species exhibit a decline from cambium toward the stem center [27,28,29,32,38,43,44]. In some woody trees, a xylem age difference of only one year can lead to significant differences in sugars and starch [31]. These general trends for woody tree species illuminate three issues in relation to my results. First, the general axial pattern is essentially reversed, with the central tissues of a woody tree stem containing the least and those of a *Cycas* stem containing the greatest concentrations of NSCs. Second, this simple sampling protocol of sampling internal distance from a fixed secondary cambium layer would not be possible within the unique cycad stem (Figure 1). However, the radial changes in NSCs within the woody trees may have been under the control of physical distance from cambium rather than xylem age per se. The pith and cortex tissues of arborescent cycad species are sizeable, and refined sampling of radial sections from the innermost vascular cylinder toward the center for pith and from the outermost vascular cylinder toward the stem periphery for cortex would improve our understanding of how NSCs are stored in a cycad stem. Third, tissue age in the arborescent cycad stem is not correlated with axial position, as secondary expansion in sub-apical stem locations occurs from random mitoses throughout all of the cycad stem’s tissues [17]. However, the oldest and original vascular cylinder is always the innermost cylinder, and the youngest vascular cylinder is always the peripheral cylinder [18]. Therefore, further research to improve our understanding of the influence of radial sections on resource storage of cycad stems could include separation in age of the vascular system by demarcating the radiating cylinders from oldest to youngest.

One caveat concerning radial sampling protocols is that dermal tissue which was included in the cortex samples may have caused a dilution of the live tissue contents. Some of the protective dermal tissue of a cycad stem is not alive. Continued research on radial zonation and stored cycad stem resources may include separation of the dermal layer from the live cortex tissues to better understand what proportion of the cortex resource pool is restricted to the live tissues and is therefore presumably available to be mobilized and deployed to ephemeral sink locations.

In closing, CAS infestations of *Cycas* plants generate depletions of NSCs [12,13]. This herbivore is the main threat to *C. micronesica* and the reason that the species is endangered [11]. Therefore, a greater understanding of how CAS decreases plant health and increases plant mortality is needed to inform critical conservation planning. An increased understanding of the means by which CAS-caused depletions of *Cycas* stem NSCs differ among all axial and radial strata is needed to inform this agenda.

## Figures and Tables

**Table 1:** The influence of radial position within *Cycas micronesica* stems on non-structural carbohydrates. Means are a combination of apical and basal stem regions.

Variable	Pith	Vascular	Cortex	Significance
Fructose (mg·g^−1^)	18.21 ± 4.34 ^b,z^	6.30 ± 1.29 ^a^	11.65 ± 1.57 ^ab^	0.0123
Glucose (mg·g^−1^)	28.83 ± 6.43 ^b^	11.30 ± 2.72 ^a^	11.98 ± 1.94 ^a^	0.0069
Maltose (mg·g^−1^)	4.16 ± 0.98 ^b^	0.11 ± 0.003 ^a^	0.10 ± 0.004 ^a^	<0.0001
Starch (mg·g^−1^)	194.69 ± 7.95 ^b^	159.05 ± 11.58 ^a^	191.06 ± 12.74 ^b^	0.0412
Total sugars (mg·g^−1^)	171.94 ± 10.51 ^c^	86.95 ± 2.26 ^a^	128.32 ± 7.29 ^b^	<0.0001

^z^ Means followed by the same letter within each row are not different according to Least Significant Difference. Mean ± SE, n = 20.

**Table 2:** The influence of axial and radial locations within *Cycas micronesica* stems on non-structural carbohydrates (NSC).

Variable	Apex Pith	Apex Vascular	Apex Cortex	Base Pith	Base Vascular	Base Cortex	Sig
Sucrose (mg·g^−1^)	116.5 ± 6.4 ^b,z^	72.5 ± 4.6 ^a^	83.7 ± 7.9 ^a^	125.0 ± 10.9 ^b^	66.0 ± 1.8 ^a^	126.8 ± 11.5 ^b^	0.0056
Total NSC (mg·g^−1^)	379.0 ± 15.4 ^c^	267.3 ± 20.3 ^ab^	295.9 ± 22.0 ^bc^	354.3 ± 10.6 ^c^	224.7 ± 13.9 ^a^	342.8 ± 19.9 ^c^	0.0211

^z^ Means followed by the same letter within each row are not different according to Least Significant Difference. Mean ± SE, n = 10.
